# Tertiary lymphoid structures in gynecological cancers: prognostic role, methods for evaluating, antitumor immunity, and induction for therapy

**DOI:** 10.3389/fonc.2023.1276907

**Published:** 2023-11-10

**Authors:** Ke Zhang, Xiao Xie, Shuang-Lin Zheng, Yuan-Run Deng, Dan Liao, Hai-Chen Yan, Xi Kang, Hui-Ping Jiang, Sui-Qun Guo

**Affiliations:** ^1^Department of Gynecology, Pingxiang People's Hospital, Pingxiang, Jiangxi, China; ^2^Department of Urology, Pingxiang People's Hospital, Pingxiang, Jiangxi, China; ^3^Department of Gynecology, The Third Hospital of Mianyang, Mianyang, Sichuan, China; ^4^Department of Obstetrics and Gynecology, The Third Affiliated Hospital, Southern Medical University, Guangzhou, Guangdong, China; ^5^The Third Clinical College, Southern Medical University, Guangzhou, Guangdong, China

**Keywords:** tertiary lymphoid structures, gynecological cancers, favorable prognosis, antitumor immunity, therapeutic induction

## Abstract

Tertiary lymphoid structures (TLSs), referred to as tertiary lymphoid organs and lymphoid tissue neogenesis, are aggregates of immune cells that occur in nonlymphoid tissues. In recent years, it has been found that TLSs within the tumor microenvironment have been associated with local adaptive immune immunity against cancer and favorable prognosis in several human solid tumors, including gynecological cancers. The issue of the prognosis of gynecological cancers, including endometrial, cervical, and ovarian cancer, is an enormous challenge that many clinical doctors and researchers are now facing. Concerning the predictive prognostic role of TLSs, effective evaluation, and quantification of TLSs in human tissues may be used to assist gynecologists in assessing the clinical outcome of gynecological cancer patients. This review summarizes the current knowledge of TLSs in gynecological cancers, mainly focusing on the potential mechanism of TLS neogenesis, methods for evaluating TLSs, their prognostic value, and their role in antitumor immune immunity. This review also discusses the new therapeutic methods currently being explored in gynecological cancers to induce the formation of TLSs.

## Background

Gynecological cancers, mainly include uterine corpus, cervical, and ovarian cancer, rank as the leading cause of female death and are an essential barrier to increasing life expectancy worldwide (1). Uterine corpus cancer is often referred to as endometrial cancer, as over 90% of cases have lesions arising in the endometrium, and its survival rate has not substantially improved since the mid-1970s (2). Ovarian cancer is the second most common cause, and cervical cancer is the fourth most common cause of cancer deaths among females worldwide (3, 4). The issue of the prognosis of gynecological cancers is an enormous challenge that many clinical doctors and researchers are now facing. In recent years, it has been found that tertiary lymphoid structures (TLSs) within the tumor microenvironment (TME) have been associated with clinical survival and outcomes in several human solid tumors (5–7).

TLSs frequently occur in many pathological conditions, such as inflammatory and infectious diseases, autoimmune diseases, and cancers (8–11). In autoimmune diseases, TLSs exacerbate local autoimmune-mediated inflammation and tissue damage, and the increased severity of the disease is associated with more or larger TLSs in the affected tissue (12–14). TLSs also develop in atherosclerotic arterial segments and correlate with plaque development (15, 16). However, TLSs, identified in several solid tumor types, are associated with enhanced immune control of cancer growth and favorable clinical outcomes (17, 18). Moreover, the presence of TLSs acts as a favorable prognostic feature in many malignant tumors, regardless of the stage of the disease (5, 19–22). Concerning the predictive prognostic role of TLSs, effective evaluation, and quantification of TLSs in human tissues may be used to assist gynecologists in assessing the clinical outcome of gynecological cancer patients.

The emergence of immunotherapy provides more opportunities for cancer treatment (23–25). However, not all cancer patients treated with immunotherapy show satisfactory response rates (26, 27), and biomarkers to select ideal patients who could benefit from immunotherapy are urgently needed. It has been reported that TLSs act as predictive biomarkers of the response to cancer immunotherapy in melanoma, soft tissue sarcoma, and renal cell carcinoma (22, 28–30). This predictive role of TLSs was independent of PD-L1 expression status and CD8+T cell-density in a large-scale retrospective analysis (31). Notably, accumulating evidence suggests that TLSs are capable of generating or enhancing adaptive immune immunity and improving the efficacy of immunotherapy in humans and mice (17). Therefore, a hypothesis that TLS induction may provide a new opportunity for cancer therapy was proposed, and it has been confirmed in mouse models of ovarian cancer that TLS induction could inhibit tumor progression (32, 33).

This review briefly summarizes the current knowledge of TLSs in gynecological cancers, mainly focusing on the potential mechanism of TLS neogenesis, methods for evaluating TLSs, their prognostic value, and their role in antitumor immune immunity. This review also discusses the new therapeutic methods currently being explored in gynecological cancers to induce the formation of TLSs.

## The potential mechanism of TLS neogenesis

**Figure 1:**
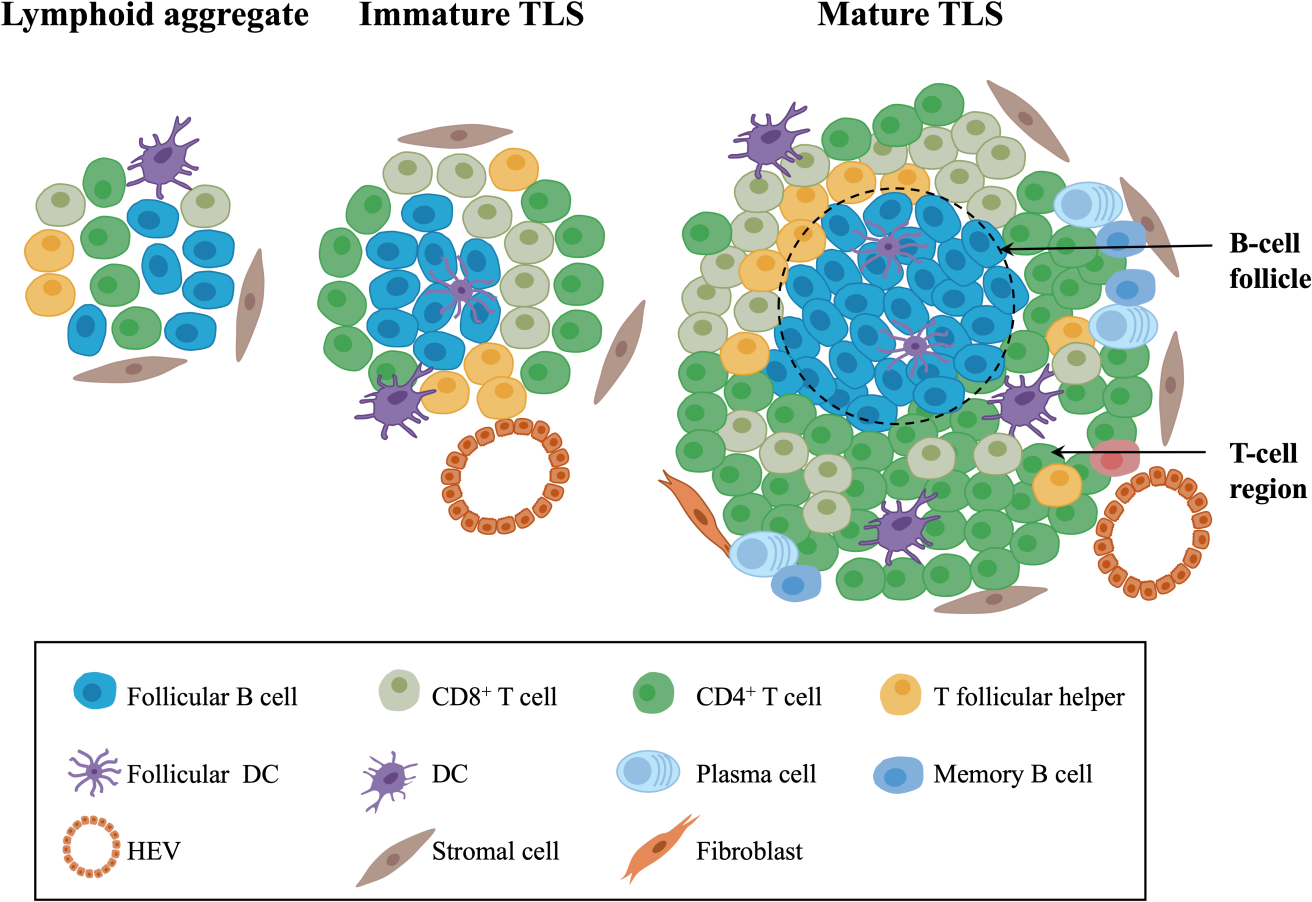
The composition of TLSs in gynecological cancers. Morphologically, TLSs are heterogeneous and vary from simple, diffuse lymphoid aggregates to dense, highly organized structures similar to lymph nodes. Mature TLSs mainly consist of B-cell follicles with typical GCs distinguished by interdigitating networks of CD21+ fDCs, adjacent T-cell regions that contain CD4+ T cells, CD8+ T cells, T follicular helper cells, and PNAd+ HEVs. In terms of cell distribution, B cells gradually occupy the center of TLSs, while the central distribution of T cells in early-stage TLSs changes to the periphery over time. TLSs, tertiary lymphoid structures; GCs, germinal centers; fDCs, follicular DCs; HEVs, high endothelial venules.

TLSs, also referred to as tertiary lymphoid organs and lymphoid tissue neogenesis, are aggregates of lymphocytes that occur in nonlymphoid tissues (17, 34). TLSs mainly consist of B-cell follicles with/without typical germinal centers (GCs) distinguished by interdigitating networks of CD21+ follicular DCs (fDCs), adjacent T-cell regions that contain CD4+ T cells and CD8+ T cells, and PNAd+ high endothelial venules (HEVs) (35–37) (**Figure 1**).

To date, the mechanism of lymphoid neogenesis in the TME remains incompletely understood. Some studies suggest a parallel relationship between the neogenesis of TLSs and SLOs in that TLS neogenesis might require the local accumulation of the homeostatic chemokines CXCL13, CCL19, CCL21, and IL-7, which mediate the migration and activation of lymphoid tissue inducer cells (LTi) (38–41). LTi cells interact with stromal organizer cells and promote the recruitment of lymphocytes and the organization of TLS structures, resulting in the induction of lymphoid neogenesis (42, 43). Unlike SLOs that form during embryogenesis, TLSs form after birth. Other molecular and cellular interactions of local chronic inflammatory stimuli also induce the neogenesis of TLSs. The accumulation of local cytokines and lymphoid chemokines and the formation of vascular structures also promote the recruitment and activation of B and T cells, which sustains the formation and assembly of TLSs (11, 44–46). Current research also suggests that neoantigens are recognized by B and T cells and can promote the neogenesis of TLSs in human tumors (47, 48). For instance, in uterine cancer, POLE-EDM and MSI tumors with a higher neoantigen burden show a significantly increased number of TLSs compared with MSS tumors, which have a lower number of mutations and neoantigens (48). In HGSOC omental metastases and esophagogastric adenocarcinoma, antigen-specific B-cell responses within TLSs include clonotype selection and expansion of B cells and somatic hypermutation and isotype switching of immunoglobulins (21, 49).

## Methods for evaluating TLSs in gynecological cancers

Concerning the favorable prognostic role of TLSs, effective evaluation, and quantification of TLSs in human tissues may be used to assist clinicians in assessing the clinical outcomes of gynecological cancer patients. It should be noted that the methods and markers used to evaluate TLSs often differ in existing studies of gynecological cancer (17, 50) (**Table 1**).

**Table 1:** Methods for Evaluating TLSs and the prognostic role of TLSs in gynecological cancers.

Primary or metastasis tumors	Cancer types	Cases	Stage	HE	IHC	Gene expression	Prognostic value	Refs
Primary cancer patients	Cervical cancer	93	I-II	–	CD20, CD3, CD21, and PNAd	–	favorable	(51)
	Endometrial cancer	378	I-III	HE	L1CAM	–	favorable	(19)
	Endometrial cancer	116	I-IV	–	CD20	–	–	(48)
	Endometrial cancer	104	I-IV	HE	–	–	favorable	(52)
	Endometrial cancer	85	–	HE	–	–	negative	(53)
	Ovarian cancer	60	–	–	CD20 and CD3	CETP, CCR7, SELL, LAMP3, CCL19, CXCL9, CXCL10, CXCL11, and CXCL13	favorable	(54)
	HGSOC	376	I-IV	HE	–	12-chemokine genes	favorable	(20)
	HGSOC	185	I-IV	HE	CD20, CD4 and CD8	–	favorable (in the presence of CXCL13)	(55)
	HGSOC	97	I-IV	HE	CD21	–	No value (PFS)	(32)
	HGSOC	81+66	I-IV	–	LAMP and CD20	–	No value (OS)	(56)
	HGSOC	30	–	–	CD4, CD8, CD20, CD21, CD208 and PNAd	–	–	(57)
mouse model	Ovarian cancer	20	–	–	CD19 and CD3	–	favorable	(33)
	Ovarian cancer	20	–	HE	–	–	favorable (along with CXCL13)	(58)
metastasis cancer patients	HGSOC omental metastases	41	III-IV	–	CD4, CD8, MECA79, Ki67 and CD45RO	–	–	(21)
Vaccinated patients	HPV16+ CIN2/3	12	–	HE	–	–	TLS neogenesis	(59)

Hematoxylin-eosin (HE) staining is the most straightforward strategy to count/quantify TLSs based on morphology. This stain is also easily obtained in clinical pathological analysis. In our previous study, TLSs were counted in HE-stained sections of HGSOC patients based on morphology (20). Nanda Horeweg et al. detected rounded aggregates of organized lymphocytes in the myometrial wall or at the tumor-invasive border as mature TLSs in endometrial cancer (19). However, TLSs counted by HE-stained sections could be potentially underestimated, easily subjected to objective bias, and poorly reproducible between pathologists (60). The literature also suggests that it is difficult to determine whether lymphocyte aggregates common in human and mouse cancers are real TLSs or just an area containing dense lymphocyte infiltration (61).

Immunohistochemistry (IHC) staining of markers of TLS-associated components, such as CD4+ and CD8+ T cells, CD20+ B cells, and PNAd+ HEVs, in consecutive tumor sections is commonly used to quantify TLSs (62). Then, quantitative digital pathological software is used to analyze the density, size, and cell content of TLSs on scanned images. Ying Zhang et al. detected the presence of TLSs in cervical cancer through IHC staining of CD20, CD3, CD21, and PNAd (51). However, research using multiparametric analysis emphasized the potential of “duplicate counting” or being hampered by section-to-section variability when studying single molecules in complex environments (63). In HGSOC, David R et al. performed multicolor IHC, which could simultaneously detect CD8, CD4, CD20, CD21, CD208, and PNAd (57). The staining results of the four types of lymphocyte aggregates also indicated that the development of TLSs followed a continuous maturation stage and ultimately produced a GC reaction. IHC staining of other biomarkers may also be used to quantify TLSs. In endometrial cancer, L1CAM was only expressed in mature TLSs with GCs, suggesting that L1CAM may serve as a specific marker for quantifying mature TLSs in clinical practice (19).

**Figure 2:**
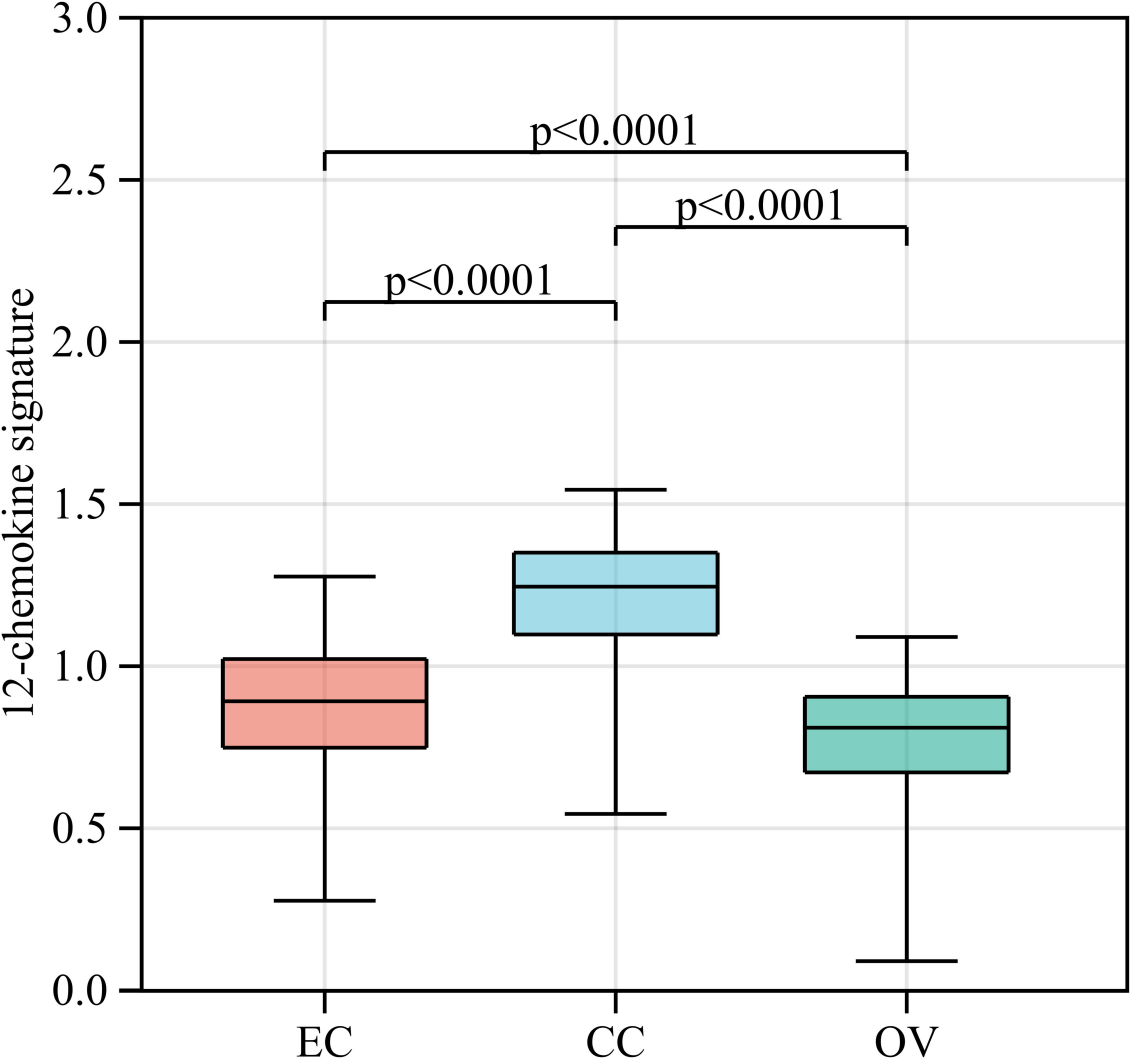
The 12-chemokine transcriptome signature of gynecological cancers. RNA-seq data of EC (n=543), CC (n=304), and OV (n=376) patients were downloaded from The Cancer Genome Atlas [https://www.portal.gdc.cancer.gov/]. The raw data (FPKM form) was transformed into the log (FPKM+1) for further analysis. Based on the RNA-seq data of gynecological cancer patients, the 12-chemokine transcriptome signature was calculated by the GSVA method (70) as processed in the Sangerbox platform [https://vip.sangerbox.com/home.html]. The 12-chemokine signature of each sample is listed in **Supplementary Table 1**. The Kruskal−Wallis test was used to compare whether there were significant differences in the 12-chemokine transcriptome signature among the three groups, and the Wilcoxon test was used to compare whether there were significant differences in the 12-chemokine transcriptome signature between each pair of groups. EC, endometrial cancer; CC, cervical cancer; OV, ovarian cancer; FPKM, fragments per kilobase million; GSVA, gene set variation analysis.

Gene signatures of TLSs, for instance, genes from multiple cell populations of TLSs and lymphoid chemokines, are used to evaluate TLSs at the transcriptome level. A 12-chemokine signature (CCL2, CCL3, CCL4, CCL5, CCL8, CCL18, CCL19, CCL21, CXCL9, CXCL10, CXCL11, and CXCL13) was initially derived from a set of genes that were biologically related to inflammation and the immune response in colorectal carcinoma (64). It is also considered highly correlated with developing and maintaining TLSs and clinical prognosis in tumors, such as CCL19, CCL21, and CXCL13, the homeostatic chemokines that likely organize TLSs (45, 65). Recent studies quantified the presence of TLSs in tumors based on the transcriptome signatures of 12 chemokines in malignant tumors, including melanoma metastases, hepatocellular carcinoma, and invasive breast cancer (20, 66–69). A bioinformatics analysis also constructed a TLS signature of ovarian cancer with prognostic value, including CXCL11, CXCL13, and CCL19, which was further validated in another ovarian cancer dataset. In this review, we quantified the abundance of TLSs in gynecological cancer based on the 12-chemokine transcriptome signature (**Figure 2**). The highest 12-chemokine signature score was observed in cervical cancer, the medium score in endometrial cancer (Kruskal−Wallis test, p < 0.0001), and the lowest score in ovarian cancer, indicating the existence of abundant TLSs in cervical and endometrial cancer, while the lowest TLSs were observed in ovarian cancer.

Collectively, the methods and markers used to evaluate TLSs often differ in existing studies of gynecological cancer. In the future, it is necessary to explore more efficient and accurate methods for evaluating TLSs in different cancer types. Although the range of cellular and molecular markers used to evaluate TLSs has been variable, significant evidence suggests that these structures are markers of good prognosis in gynecological cancer.

## TLSs: a prognostic biomarker in gynecology cancers

Accumulating studies indicate that the presence of TLSs is associated with clinical prognosis in gynecological cancers. In cervical cancer, patients with mature TLSs show significantly higher overall survival rates than those with no or early TLSs (51). The number of TLSs also correlates with the depth of tumor invasion, preoperative chemotherapy, human papillomavirus (HPV) infection, and high levels of PD-1 (51). In endometrial cancer, both the number and presence of TLSs are strongly related to the reduction in recurrence risk, and TLSs act as a significantly favorable predictor of recurrence, independent of other clinicopathological and molecular factors (19). In ovarian cancer, the TLSs-positive group showed better overall and progression-free survival rates than the TLSs-negative group (54). TLSs in mouse ovarian cancer models can also inhibit tumor growth (33). In high-grade serous ovarian cancer (HGSOC), the coexistence of CD8+ T cells and the B-cell lineage significantly improves the clinical prognosis and is related to the presence of TLSs (71). Another study indicated that the combination of TLSs and CXCL13 is associated with prolonged overall survival rates in HGSOC patients (55).

There is a hypothesis that TLS location may affect its prognostic value in tumors. As reported in hepatocellular carcinoma, intratumoral TLSs reflect the existence of ongoing, effective antitumor immunity and are associated with a lower risk of early relapse (5), while TLSs in nonneoplastic liver tissue promote tumor development and are associated with adverse outcomes (67). However, in gynecological cancer, there is limited exploration of the relationship between TLSs at different locations and prognosis. Only a single-center, retrospective cohort study of type II endometrial cancer found that peritumoral lymphocytes with band-like structures in the forefront of endometrial cancer are associated with fatal outcomes (19). Therefore, it is necessary to conduct more detailed research on the location of TLSs in gynecology cancers to explore the relationship between TLS location and prognosis. Collectively, TLSs could serve as a novel predictive biomarker to stratify the overall survival risk of gynecology cancer patients.

## Role of TLSs in the regulation of immune responses

**Figure 3:**
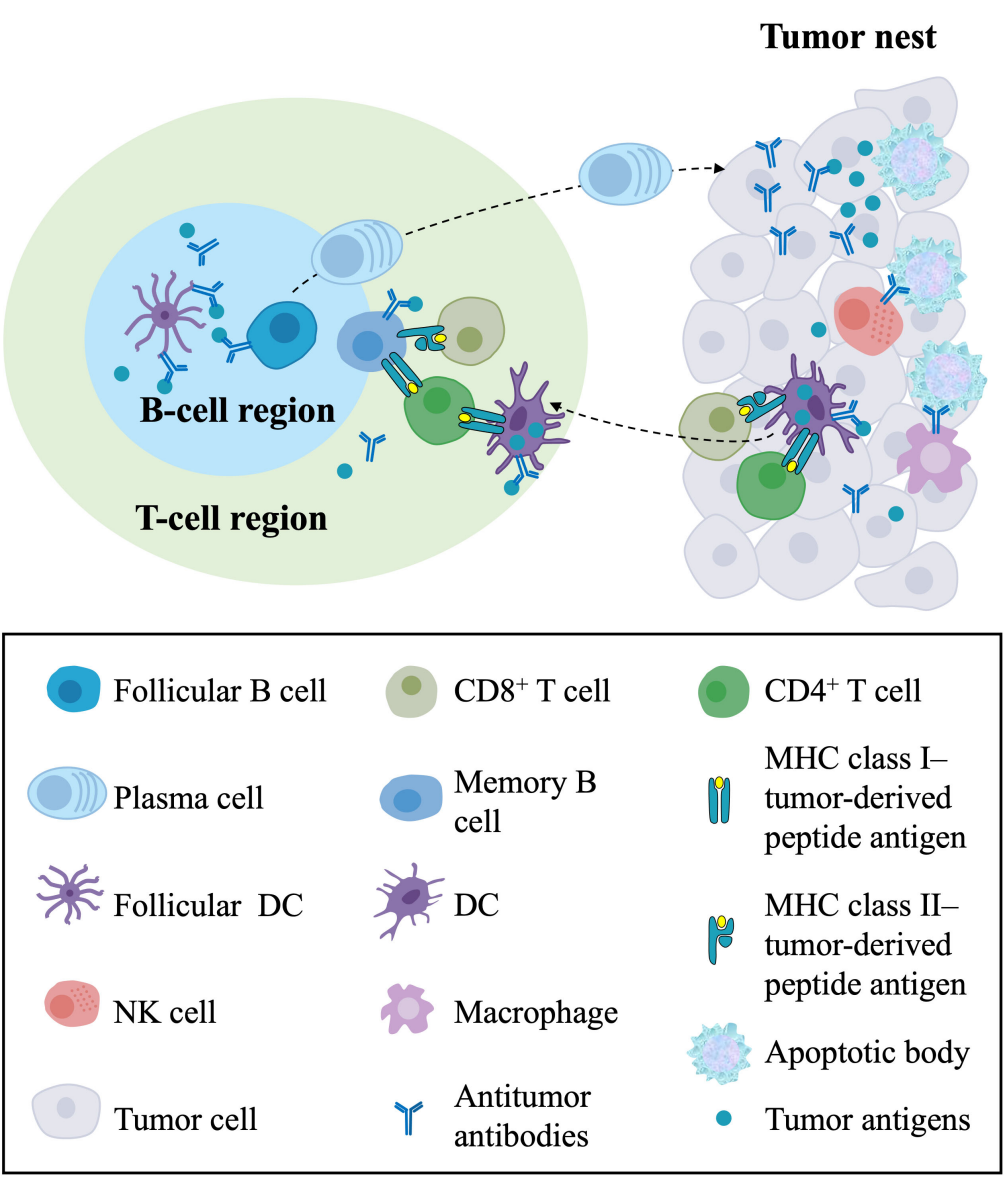
The antitumor immune responses of TLSs and their components in the TME. In the T-cell region of TLSs, DCs capture and process tumor antigens and present the processed antigens to CD4+ and CD8+ T cells, resulting in the priming and activation of effector T-cell responses against tumors. In the B-cell region of TLSs, follicular DCs present tumor antigens in the form of immune complexes to follicular B cells, leading to the proliferation and activation of B cells and the differentiation of memory B cells and plasma cells. Plasma cells migrate to the tumor nest and produce antitumor antibodies, directly forming immune complexes with tumor antigens and leading to ADCC and opsonization. In the tumor nest, CD8+ T cells directly mediate cytolytic activity against tumors, and NK cells and macrophages with Fc receptors kill target tumor cells by recognizing the Fc segment of the antibody. In addition, the killing of cancer cells releases tumor antigens that can be captured and processed by DCs in the TME, which migrate to TLSs. TLSs, tertiary lymphoid structures; TME, tumor microenvironment; DCs, dendritic cells; ADCC, antibody-dependent cellular cytotoxicity; NK cell, natural killer cell.

The generation and regulation of adaptive immune responses against cancers generally occur in secondary lymphoid organs (SLOs), such as regional lymph nodes and the spleen (72–75). Further research on the TME has found that TLSs represent the local initiation and expansion sites of antigen-specific T and B cells directly in tumor tissues, indicating that antitumor adaptive immunity also occurs in TLSs (7, 36, 76, 77) (**Figure 3**). Here, we summarize the antitumor immune effects of TLSs in ovarian cancer. TLSs represent well-organized clusters of tumor-infiltrating lymphocytes, mainly consisting of B-cell follicles, adjacent T-cell regions, and PNAd+ HEVs (35, 36). The previous consensus of the field was that T cells are the primary mediator of antitumor immunity, as CD8+ T cells can directly mediate cytolytic activity against tumors (78, 79). However, it has been found in human and mouse tumors that the prominent component of TLSs is B-cell follicles. Recently, the contribution of B cells to the antitumor immune response has been increasingly investigated. B cells have been associated with a better clinical prognosis in human primary and metastatic ovarian cancer (21, 80, 81). B cells in GCs within mature TLSs can undergo clonotype selection, expansion, somatic hypermutation, affinity maturation, and isotype switching, suggesting active humoral antitumor immunity in these structures (71). Then, these B-cell clones differentiate into plasma cells, also known as effector B cells, that can produce mature, oligoclonal IgG transcripts, potentially leading to antibody-dependent cellular cytotoxicity (ADCC) and opsonization (57). A strong B-cell memory response within TLSs found in HGSOC omental metastases, including a restricted repertoire of antigens and production of tumor-specific IgG transcripts by plasma cells, supported the development of antitumor immunity (21). It has also been found that immune complexes with IgG transcripts promote CD86 expression on *in vitro*-generated APCs, suggesting that IgG transcripts might promote antitumor responses by enhancing DC priming (21). These findings suggest that B-cell differentiation and the production and accumulation of antitumor antibodies are critical components of the effective antitumor immunity of TLSs.

In autoimmune diseases, infiltrating B cells can serve as effective APCs to initiate and activate T cells, thereby influencing and regulating autoimmune processes (82–84). Consistent with this, in primary HGSOC, CD20+ B cells express costimulatory molecules associated with APCs, such as CD80, CD86, CD40, MHC class I, and MHC class II (71). B cells are also involved in recruiting and supporting DCs and neutrophils in TLSs of omental metastases by secreting CXCL8 (21). These findings suggest that B cells may enhance cellular immunity against tumors in an APC-dependent manner. In addition, it has previously been shown in many studies that CD20+ B cells and CD8+ T cells often colocalize in TLSs within and adjacent to tumor islets (21, 57, 71) and significantly improve the prognosis of HGSOC. Another study of primary HGSOC found that CD8+ T cells carried prognostic benefits with the presence of PCs, CD20+ B cells, and CD4+ T cells (57). Therefore, a novel cooperative interaction between B cells and T cells that reflects a supportive role of B cells in cytolytic immune responses is proposed (71, 80). As sites for the infiltration and proliferation of T and B cells, TLSs orchestrate cellular and humoral immunity to promote a synergistic antitumor immune response.

Although most research focuses on T and B cells in TLSs, other components also play a role in antitumor immunity—for example, CD21+ DCs and PNAd+ HEVs. The majority of mature DCs were localized in the stroma of the tumor and associated with TLSs. DCs in TLSs contribute to the local initiation/activation of T-cell dependent antitumor immunity, characterized by TH1 polarization and cytotoxic functions, and correlate with improved prognosis in HGSOC patients (56). CD21+ DCs in mature TLSs can also produce CXCL13, which is known to recruit and activate immune cells and promote the formation of TLSs in tumors (32). As the characteristic component that is only found within TLSs in tumors (45), PNAd+ HEVs play a critical role in orchestrating antitumor immunity, as they mediate both the type of lymphocyte and the site of entry into lymphoid tissues through the expression of vascular addressins (85). PNAd+ HEVs are also associated with an improved prognosis in many cancers (86–88) and are thought to actively modulate antitumor immune activity.

Taken together, TLSs create an organized immune structure where tumor-infiltrating B and T cells and DCs interact and activate each other, promoting a local sustained immune response, and TLSs promote the cooperative antitumor response of cellular and humoral immunity.

## The induction of TLSs in gynecological cancer therapy

Since TLSs represent the main site of the immune response against tumors in the TME, inducing TLSs as an immunomodulatory target to enhance antitumor immunity seems to be a new strategy for gynecological cancer therapy. To date, research on the induction of TLSs in cervical and endometrial cancer has been limited. In contrast, the feasibility of local TLS induction has been explored in murine models of ovarian cancer. Immunocompetent mouse recombinant CXCL13 showed a significantly increased area of TLSs per tumor area, increased infiltration of CD8+ T cells around TLSs, and prolonged survival time compared with the control group (32). In contrast, CXCL13 blockade abrogated TLS formation in intraperitoneal tumor-bearing mice, increasing tumor growth (33). The literature also found that CXCL13 shapes an immunoreactive TME by facilitating the maintenance of CXCR5+CD8+ T cells in TLSs, and the combination of CXCL13 and anti-PD-1 significantly inhibited tumor growth in subcutaneous murine models of ovarian cancer (55).

Other immunotherapies can also induce the formation of TLSs in murine models of ovarian cancer. T follicular helper (Tfh) cell differentiation promoted by silencing Satb1 was shown to be sufficient to drive TLS assembly in mouse models of ovarian cancer, and mice with TLSs induced by Tfh cell differentiation showed decreased tumor growth (33). Combining an oral FAK inhibitor with TIGIT-blocking antibody immunotherapy increased TILs and CXCL13 levels, leading to TLS formation and prolonged survival in experimental mouse models of aggressive ovarian cancer (58). Moreover, the cancer vaccine was shown to promote TLS formation in cervical intraepithelial neoplasia (CIN2/3) lesions. Patients with high-grade CIN2/3 showed induced TLSs and increased infiltration of tumor-infiltrating lymphocytes after undergoing peripheral vaccination with HPV antigens (59). Our previous research speculated that TLSs are also associated with a good response to immune checkpoint block therapy in HGSOC patients. However, clinical samples are lacking to validate this inference (20). These data support the strategy of inducing TLSs to improve the efficacy of antitumor responses and provide more opportunities to control and treat gynecological cancer in the future.

As mentioned above, TLSs are usually associated with increased disease severity and adverse prognostic outcomes in many cases of autoimmunity (12–14). Inducing TLSs enhances antitumor immunity and enhances autoimmune B and T-cell responses in other parts, which are detrimental to autoimmunity. However, there are limited data on the role of TLSs in immune-related adverse events. Therefore, further exploration of the potential of TLS induction is necessary to enhance antitumor immunity and affect autoreactive T and B cells. The risk-benefit ratio of this method needs to be carefully evaluated in the future.

## Concluding remarks

In conclusion, TLSs serve as a novel predictive biomarker to stratify the overall survival risk of gynecology cancer patients. Although the range of cellular and molecular markers used to evaluate TLSs has been variable, significant evidence suggests that these structures are markers of good prognosis in gynecological cancers. TLSs serve as local sites for the presentation of tumor antigens by DCs and the expansion and activation of tumor-infiltrating T and B cells, resulting in tumor antigen-specific cytotoxic T cells and the production of antibodies by plasma cells. Regarding cancer therapy, ovarian cancer studies have found that inducing TLS formation through various methods, such as immunotherapy, can inhibit tumor growth. Meanwhile, the cancer vaccine can also induce the formation of TLSs in CIN2/3 patients. Therefore, the induction of TLSs may provide more opportunities to control and treat gynecological cancers in the future.

## Author contributions

KZ: Conceptualization, Investigation, Writing – original draft, Writing – review & editing. XX: Conceptualization, Investigation, Writing – original draft, Writing – review & editing. S-LZ: Formal Analysis, Visualization, Writing – original draft. Y-RD: Formal Analysis, Funding acquisition, Visualization, Writing – original draft. DL: Formal Analysis, Visualization, Writing – original draft. S-QG: Methodology, Supervision, Writing – review & editing. H-PJ: Methodology, Supervision, Writing – review & editing. H-CY: Writing – original draft, Visualization. XK: Writing – original draft, Visualization.
